# The effect of a flexible thoracolumbar brace on neuromuscular scoliosis: A prospective observational study

**DOI:** 10.1097/MD.0000000000026822

**Published:** 2021-08-13

**Authors:** Joonyoung Jang, Yulhyun Park, Seungeun Lee, Seon Cho, Jun Chang Lee, Sunmok Hong, Jiwoon Lim, Ju Seok Ryu

**Affiliations:** a Department of Rehabilitation Medicine, Seoul National University Bundang Hospital, Seongnam; b Department of Rehabilitation Medicine, Seoul National University College of Medicine; c Department of Rehabilitation Medicine, Seoul National University College of Medicine, Seoul National University Hospital, Seoul, South Korea.

**Keywords:** brace, cerebral palsy, neuromuscular diseases, orthotic devices, scoliosis

## Abstract

Neuromuscular scoliosis is a common deformity seen in patients with neuromuscular diseases. Although rigid thoracolumbosacral orthosis is the most frequently used brace, it has low compliance rates and can lead to complications including skin ulcers. Thus, alternative methods for treating neuromuscular scoliosis are needed. The purpose of this study is to evaluate the clinical effects of a novel flexible brace to prevent the progression of neuromuscular scoliosis.

This study is a prospective observational study. Twenty-three patients with neuromuscular scoliosis were enrolled in the study. Among patients diagnosed with neuromuscular disease, spine radiographs were checked for a neuromuscular scoliosis diagnosis. The participants were treated with a novel flexible brace for 6 months. The control group (n = 46) was selected using propensity score matching method from a clinical data warehouse. The Cobb angle was measured and compared between the study and control groups.

In the study group, the average Cobb's angle significantly decreased from 47.22 ± 18.9° to 31.8 ± 20.0 when wearing the flexible brace (*P* < .001). Thus, the correction rate was 36.9%. The annual progression rate was significantly lower in the study group than in the control group (*P* *<* .05).

The flexible brace showed a significant correction rate of scoliosis in patients with severe neuromuscular diseases. The flexible brace is an alternative treatment modality for patients with neuromuscular scoliosis. Daily application of the flexible brace during the growing period can reduce the degree of fixed deformity in the long term.

## 1 Introduction

Neuromuscular scoliosis is a common deformity seen in patients with one or several of a large number of neuromuscular diseases (NMD), including cerebral palsy (CP), encephalopathy, poliomyelitis, myelomeningocele, spinal muscle atrophy, muscular dystrophies, and myopathies.^[1,2]^ The reported incidence of neuromuscular scoliosis varies greatly from 15% to 80% and differs based on patient characteristics.^[3]^ Scoliosis rapidly progresses from a flexible form to a fixed deformity during the growth period in children with NMD, and it continues to progress even after the child's growth has ended.^[4]^

Scoliosis causes problems with sitting, which influence vision, communication, mobility, feeding, and pulmonary function, and can lead to pressure sores and pain in children with NMD.^[3,5,6]^ Surgical treatment methods have been successful, even in cases of severe scoliosis. However, surgery is not without the risk of complications, and many patients are poor candidates for major surgery due to the presence of coexisting medical diseases.^[7]^ Complications occur in 40% to 80% of patients who undergo spine fusion, with death occurring in approximately 1%.^[3,8,9]^ Thus, a safe and reliable orthotic treatment method is essential for these patients.^[10]^

In 2000, Terjesen et al^[11]^ reported that patients with scoliosis who were treated with a rigid thoracolumbosacral orthosis (TLSO) had an annual progression rate (APR) of 4.2°. However, their study did not include a control group and was retrospective in nature, which is prone to bias. Although rigid TLSO is the most frequently used brace, it has low compliance rates and can lead to complications including skin ulcers. Furthermore, the rigid TLSO brace must be replaced frequently as children grow.

The current study hypothesized that increasing the compliance rate of a brace in patients with NMD during the growth period may help reduce the severity of scoliosis. The conventional rigid brace was exchanged for a flexible form brace to decrease complications and improve compliance. This study had 2 purposes: to evaluate the clinical effects of a novel flexible brace to prevent the progression of scoliosis in patients, and to determine the rates of satisfaction and complications with the flexible brace in these patients.

## 2 Methods

### 2.1 Study design and participants

This prospective, single-center, open-label clinical trial took place from August 2018 to February 2020. The study protocol was approved by the institutional review board of the hospital (B-1805/471-003, B-1807/481-005) and registered on clinicaltrials.gov (Registration number: NCT04012125, NCT04012112). All patients or their legal guardians provided written informed consent before participation. Data and safety were monitored every 6 months.

Two separate studies for CP and other NMD were planned. The previously described methods were used to calculate the required sample size.^[11]^ The average Cobb angle of patients before enrollment was 68.4° ± 24.0°, and the average Cobb angle with the brace was 43.9° ± 21.9°. For an α<0.05 in 2-tailed tests and a power of 80%, the target sample size required was 10 patients. However, 14 patients with CP were selected due to a predicted dropout rate of 20% and potential outlier data, and 12 patients with NMD were selected due to a predicted dropout rate of 10% and the number of available patients.

During the screening process for the study group, researchers recorded demographic data, medical history, and drug history, and obtained written informed consent for each patient. Each patient underwent a thorough physical examination. Before any interventions, radiography was performed to check the whole spine [anteroposterior (AP) and lateral in the sitting position, and bilateral bending in the supine position]. The bilateral bending images were used to define rigid scoliosis.

For the study group, eligible participants were aged 3 to 15 years with a diagnosis of CP; a Gross Motor Function Classification System (GMFCS) level of III, IV, or V; and an initial Cobb angle between 20° and 45°. Patients with a Cobb angle >45° who declined surgery were also included. The diagnosis and classification of CP were made in accordance with the international consensus.^[12]^ Motor function was recorded in accordance with the GMFCS.^[13]^ The exclusion criteria were a history of spine surgery, Cobb angle < 20°, living in a protective facility, rigid scoliosis (i.e., change in the Cobb angle of <10°), other NMDs, and refusal to provide informed consent.

**Figure 1:**
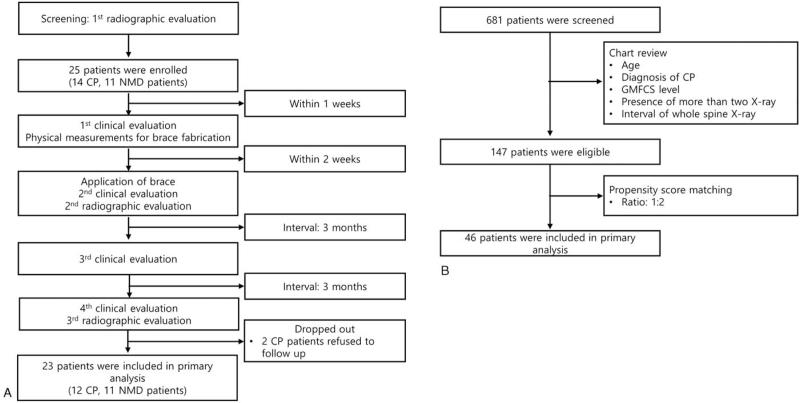
Enrollment process and flow chart of the study design. (A) Patients underwent a pre-enrollment screening, at which time baseline radiographs were obtained. Twenty-five patients were enrolled in the study and underwent the first clinical evaluation. Measurements for a custom-made brace were taken at this time. The braces were delivered to the patient within 2 weeks of the first evaluation. At that time, a second clinical evaluation was performed, and radiographs of the patient wearing the brace were obtained. Three months later, the patients underwent a third clinical evaluation. After another 3 months, patients underwent the final clinical evaluation, and final radiographs were obtained. Two patients were lost to follow-up. (B) Using the hospital's clinical data warehouse, a historical control group was formed. Out of 681 cases in the database, 147 met the study eligibility requirements. From those 147 patients, propensity score matching was used to create a control group of 46 patients.

Twenty-five eligible participants were enrolled in the study group. Two CP patients were lost to follow-up and the remaining 23 patients were included in the primary analysis (Fig. 1A). A control group was formed using the hospital's clinical data warehouse program. A total of 681 patients were selected using the search criteria of age, sex, diagnosis of CP, and more than 2 radiographic tests between July 2010 and July 2019, and 147 of these patients met the eligibility requirements of the study. Propensity score matching was used to select 46 patients based on age, gender, GMFCS, and Cobb angle (Fig. 1B).

### 2.2 Procedures

Before the first clinical evaluation, each patient underwent screening for eligibility and their first radiographic evaluation. Physical measurements were obtained at the first clinical evaluation in order to create the flexible brace. Immediately after the brace was provided to the patient, a second clinical evaluation and radiography were performed with the patient wearing the brace. Three months later, the third evaluation was completed. The final evaluation and the final radiography assessment were performed three months after the third evaluation. The participants were treated with the flexible brace for a total of 6 months.

Clinical evaluations included a test for pain intensity using a visual analogue scale (VAS, 0–10), the caregiver's satisfaction with the brace using a Likert scale (1–5), and quality of life questionnaires (Caregiver Priorities & Child Health Index of Life with Disabilities (CPCHILD), the Assessment of Caregiver Experience with Neuromuscular Disease (ACEND), and Muscular Dystrophy Spine Questionnaire (MDSQ)). The initial assessment of the level of satisfaction with the brace evaluated the satisfaction with the previous brace used by the patient as well as that with the flexible brace. The CPCHILD questionnaire is a validated, caregiver proxy, disease-specific instrument designed for use in children with severe CP (GMFCS levels IV and V).^[14]^ The ACEND questionnaire assesses the burden of caring for a child diagnosed with a severe NMD. The questionnaire consists of 41 items divided into 2 domains and 7 subdomains, and scores range from 0 to 100.^[15]^ The MDSQ is a valid and reliable questionnaire designed to assess the outcomes of treatment in children with muscular dystrophy and scoliosis.^[16]^

The flexible brace (Flexpine^a^) was custom made for each patient to meet their physical measurements and was based on whole spine radiographs. The physical measurements included chest circumference at the axillary and nipple levels, waist circumference, mid-pelvic circumference, and the distance between the axilla and mid-pelvis. The flexible brace was manufactured and delivered within 2 weeks of the physical measurement assessment.

For radiographic evaluations, a specialized, self-made chair was used for patients who could not sit independently. This chair had a reclined back, a seat, and a chest strap, which prevented the patient from slipping down while not affecting the degree of scoliosis. A whole spine radiograph was used to measure the Cobb angle. The radiographs obtained immediately after the patient began wearing flexible brace were used to determine if the locations of the straps were appropriate and whether the brace was correctly made. If the brace was made incorrectly, the brace was re-made and evaluated on the patient again. Patients did not wear the braces during the third set of radiographs. The Cobb angle change between the first and second set of radiographs was used to measure the correction rate. The Cobb angle change between the first and third set of radiographs was used to calculate the APR and to determine whether the flexible brace improved the natural progressive course of neuromuscular scoliosis.

The primary outcome was the correction rate of the Cobb angle, which reflects the effectiveness of the brace. The secondary outcomes evaluated the patients’ tolerance for the brace, which included the APR of scoliosis compared with that of the control group, complications of wearing the brace, pain intensity, brace satisfaction, and quality of life questionnaires results. The caregivers were instructed to have the patient wear the brace for at least 18 hours a day. In cases wherein wearing the brace for 18 hours a day was not possible, the patients were permitted to wear it for as long as possible, and caregivers were asked to record the total wearing time using a daily diary to record the wearing time.

### 2.3 Equipment

**Figure 2:**
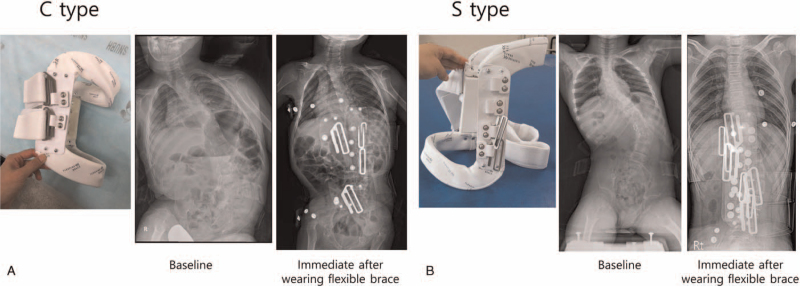
The shape of a flexible brace and the changes in the Cobb angle while wearing the brace. (A) The C-shaped flexible brace consisted of a frame and a pair of elastic straps. The C-shaped brace was used for a single curve. (B) The S-shaped flexible brace consisted of a frame and two pairs of elastic straps. This brace was used for a double curve.

The frames of the flexible braces were C- or S-shaped, depending on the location of the apex and the curve patterns. The braces were composed of a frame and elastic straps. The C-type brace had a pair of straps on the convex side, while the S-type brace had 2 pairs of elastic straps, 1 on each convex side. The lowest part of the frame aligned with the upper lateral ilium (below the iliac crest line) and acted as a fixed point (Fig. 2). The straps were located at the apex of the curves. The braces were made by manufacturers with experience in creating spinal braces for patients with neuromuscular scoliosis. The braces were replaced if they became too small or worn out.

### 2.4 Statistical analysis

Propensity score matching was used to match the study group with a historical control group.^[17]^ All statistical analyses were conducted using SPSS 25.0 software (SPSS Inc., Chicago, IL). The Wilcoxon signed rank test was used to compare the Cobb's angles at different time points in the study. The Mann–Whitney *U* test was used to compare characteristics and changes of the Cobb angles between the study and control groups. A *P* value <.05 was considered statistically significant.

## 3 Results

The demographic data are presented in Table 1. In the study group, 13 patients (56.6%) were females, and the average age was 10.1 ± 4.2 years. The average initial Cobb angle was 47.2 ± 18.9°. The average daily wearing time was 7.6 ± 5.5 hours. As propensity score matching was used, there were no significant differences between the study and control groups.

**Table 1:** Demographics and clinical characteristics.

	Study group (n = 23)	Cerebral palsy (n = 12)	NMD (n = 11)	Control group (n = 46)	*P*
Age, yr	10.1 ± 4.2	10.6 ± 3.8	9.6 ± 4.7	10.5 ± 4.3	.740
Sex (M/F)	10: 13	6: 6	4: 7	20: 26	1.000
GMFCS level (IV, V)	6: 17	3: 9	3: 8	12: 34	1.000
Cobb angle, Baseline	47.2 ± 18.9	51.2 ± 19.3	42.9 ± 18.3	49.5 ± 28.1	1.000
Cobb's angle, Follow-up	45.9 ± 22.8	51.1 ± 25.8	40.3 ± 18.7	54.8 ± 29.3	.249
Difference of Cobb angle	–1.3 ± 10.2	–0.1 ± 12.9	–2.6 ± 6.6	5.3 ± 12.3	.034^∗^
Height, cm	114.5 ± 27.1	111.2 ± 22.8	118.2 ± 31.9		–
Weight, kg	23.4 ± 15.0	19.7 ± 8.9	27.4 ± 19.3		–
Wearing time, h	7.3 ± 5.5	7.0 ± 5.9	7.6 ± 5.5		
Follow-up period, mo	6.7 ± 1.1	6.5 ± 1.1	7.0 ± 1.0	10.2 ± 5.9	.034

Table 2 summarizes the changes observed in the study group after wearing the flexible brace. The average Cobb angle of the study group significantly decreased from 47.2 ± 18.9° to 31.8 ± 20.0° while wearing the flexible brace (*P* < .001). The calculated correction rate in the study group was 36.9%. The Cobb angles of the CP and NMD subgroups within the study group significantly decreased with the flexible brace (*P* < .01 and *P* < .05, respectively). After 6 months, the average Cobb angle was 45.9 ± 22.8°, which did not significantly differ from the Cobb angle at baseline. The VAS score significantly increased after wearing the brace, but showed a decreasing tendency 6 months after the brace was introduced. There were no significant improvements in the CPCHILD and ACEND scores in the CP subgroup or in the MDSQ score in the NMD subgroup.

**Table 2:** Changes of the Cobb angle, VAS, Likert, and quality of life questionnaire after wearing FLEXpine.

	Pre-enrollment	Baseline	After Wearing	6Mo F/U
Study group				
Cobb angle, °	35.2 ± 13.3	47.2 ± 18.9	31.8 ± 20.0^∗^	45.9 ± 22.8
VAS		0.6 ± 2.0	3.7 ± 3.1^∗^	2.83 ± 3.56
Likert		3.4 ± 0.9	3.9 ± 0.5	3.5 ± 1.1
CP subgroup				
Cobb angle, °	38.6 ± 15.5	51.1 ± 19.3	32.2 ± 20.4^∗^	51.0 ± 25.8
CPCHILD		26.1 ± 16.6		27.33 ± 17.35
ACEND		37.6 ± 11.2		38.81 ± 11.12
NMD subgroup				
Cobb angle, °	30.9 ± 9.1	42.9 ± 18.3	31.3 ± 20.6^∗^	40.3 ± 18.7
MDSQ		53.1 ± 19.8		53.3 ± 20.3

When the Cobb angle at the follow-up evaluation was compared between the study and control groups, no significant difference was found. However, the Cobb angle differences between the two groups were significant (*P* < .05, Table 1).

**Figure 3:**
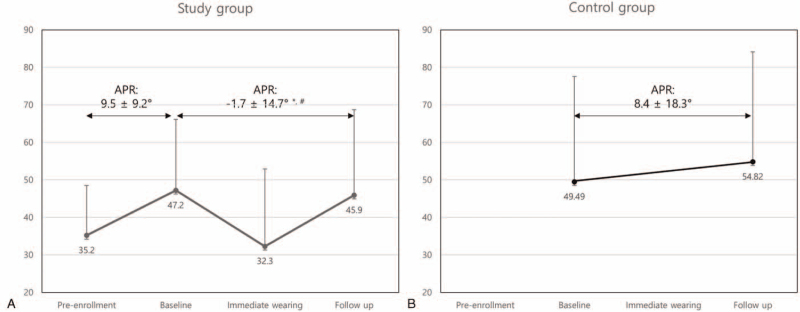
Annual progression rates of the Cobb angle. Although the annual progression rate did not change significantly, it was markedly reduced in the study group compared to that during the pre-enrollment period (A) and that in the control group (B). APR = Annual progression rate. ^∗^*P* < .05 in comparison to the pre-enrollment period. ^†^*P* < .05 in comparison to the control group.

The APR of the study group during the pre-enrollment period was 9.5 ± 9.2°. The APR significantly reduced to -1.7 ± 14.7° after 6 months of wearing the flexible brace (*P* < .05). The APR was significantly lower in the study group than in the control group (*P* < .05, Fig. 3).

One patient reported a skin ulcer that resolved after the brace was modified. No other complications were reported.

## 4 Discussion

In this study, the primary outcome was the correction rate, which reflects the effectiveness of the flexible brace. As the flexible brace was expected to have a high compliance rate compared to the conventional rigid brace, the main purpose of the study was to determine how much correction could be obtained using the flexible brace. In this study, the correction rate of the brace was 36.9% in the study group. The correction rate was not significantly different between the CP and NMD subgroups (*P* = .288). Previous studies reported that the rigid brace corrected the Cobb angle from 68.4° to 43.9° and that another dynamic spinal brace corrected the Cobb angle from 41.9° to 36.7°.^[11,18]^ Although these results cannot be directly compared with those of previous studies, the flexible brace showed satisfactory effectiveness for the correction of the Cobb angle. The advantage of this flexible brace is that it can be made to fit the patient more tightly. As the flexibility of the patient's scoliosis improves, the brace can be adjusted, suggesting that the correction rate can increase with more than 6 months of use.

Before study enrollment, the APRs of the study and control groups were 9.5 ± 9.2° and 8.4 ± 18.3°, respectively. The APR after 6 months of wearing the flexible brace was -1.7 ± 14.7° in the study group, and this was significantly lower than that during the pre-enrollment period or that of the control group. Previous studies reported that the APR in patients younger than 15 years is 4.5° to 5.4° and that it increases to 9.2° in patients with kyphoscoliosis.^[4,11,19]^

In this study, the APR of the control and study groups during the pre-enrollment period were higher than those reported in previous studies using rigid TLSO.^[4,5,11]^ The reason may be due to patients not using the brace or the severity of scoliosis, as defined by a high GMFCS level. Normally, researchers do not prescribe rigid braces for patients with neuromuscular scoliosis due to the low compliance rate, the high rate of skin complications, and the continual growth of the patients. There are several reports on the ineffectiveness of rigid braces for patients with neuromuscular scoliosis.^[5]^ The incidence and severity of scoliosis are directly related to the GMFCS level.^[13]^ In this study, only severe CP or non-ambulatory NMD patients (i.e., GMFCS level IV and V) were included; thus, the data may reflect the APR of patients with severe, restricting NMDs who do not use a brace. Although this study included patients with severe NMD, the flexible brace decreased the APR to 0.3 ± 17.7°.

An important requirement to improve the effectiveness of the brace is compliance. Patients reported wearing the brace for a shortened amount of time due to skin pressure irritation and increasing respiratory problems.^[5,11]^ To increase compliance, the flexible brace must be made and fitted properly with the goal of decreasing skin irritation. In this study, only 1 patient developed a skin ulcer; no other complications were reported. Patients tolerated the flexible brace well compared with the results of a previous study in which nearly one-third of patients using a rigid brace complained of skin problems.^[11]^ In the current study, the flexible brace was well tolerated, which is possibly due to the design of the frame and the natural characteristics of the elastic straps that allow for good ventilation, making the brace comfortable even during the summer. In addition, the elastic straps are adjustable and can be tightened or loosened according to each patient's condition. The flexible brace is easy to put on and remove and is comfortable to wear. The frame is adjustable, can be designed for patients with a wide range of physical characteristics, and can be adjusted as a patient grows. Previous reports of low compliance rates for rigid braces may be due to ill-fitting braces. Of note, patients who did not undergo surgery tolerated their spinal orthoses well.^[11]^

The main objective of the management of neuromuscular scoliosis is to maintain or improve the patients’ functional abilities and quality of life.^[20]^ This study evaluated the quality of life using the CPCHILD and ACEND questionnaires in CP patients and the MDSQ in NMD patients. The results of these questionnaires were not significantly different after using the flexible brace. This may be because the patients included in this study were not able to communicate, and only 5 of 23 patients had previously used a rigid TLSO device. Most of the caregivers in this study did not understand the discomfort of the rigid TLSO, and the pain intensity significantly increased from 0.58 ± 2.02 to 3.67 ± 3.31 after wearing the flexible brace. This may have influenced the caregivers’ perception of the ineffectiveness of the flexible brace to improve the patients’ quality of life.

The average wearing time of the flexible brace was 7.3 ± 5.5 hours, which was much shorter than the recommended wearing time of the TLSO device. Using a brace to treat NMDs differs from using a brace to treat idiopathic scoliosis, which progresses over 3 to 5 years. Therefore, it is possible for these patients to wear a brace for as long as possible. However, neuromuscular scoliosis continually worsens. At the early stage of scoliosis, the curve is flexible and easily straightened by the brace. As the patients grow, the flexible scoliosis changes to a fixed deformity, rendering a brace ineffective to correct the Cobb angle. Patients with neuromuscular scoliosis should wear a brace for their entire life. Thus, wearing the brace for more than 18 hours a day is not possible.

There are some limitations of this study. First, the sample size was too small, and only 6 months of follow up were performed to generalize the long-term effects of the brace. Second, this was not a randomized controlled trial. However, propensity score matching was used to construct a historical control group with the goal of minimizing the selection bias. Third, this study enrolled only CP patients as a control group because there were not enough NMD patients to include as the control group. However, there was no significant difference between NMD and CP patients, and the clinical course of scoliosis is similar, so the effect on the results is thought to be minimal. A future randomized, controlled, prospective study with a larger sample size and longer period of follow-up period is required.

## 5 Conclusion

The flexible brace showed a significant correction rate of scoliosis in patients with severe NMDs. The APR of the study group was significantly lower than that seen during the pre-enrollment period or in the control group. In addition, the flexible brace was easier to wear than the rigid brace and showed negligible complications. Unlike patients with idiopathic scoliosis who need only about 3 years of wearing a brace, patients with neuromuscular scoliosis must wear a brace for a longer period of time since scoliosis becomes worse, even after growth has ended. The conventional rigid brace is inconvenient to wear for a long time due to issues of ventilation, sweating, heat, and so on. Therefore, the flexible brace could be a simple and effective alternative treatment modality for neuromuscular scoliosis. Daily application of the flexible brace during the growing period can reduce the degree of fixed deformity in the long term. Further studies are required to determine the long-term effects of the flexible brace.

## Acknowledgments

The authors thank the Medical Research Collaborating Center at Seoul National University Bundang Hospital for statistical analyses.

## Author contributions

**Conceptualization:** Ju Seok Ryu.

**Data curation:** Yulhyun Park, Seungeun Lee, Seon Cho.

**Formal analysis:** Jun Chang Lee, Ju Seok Ryu.

**Investigation:** Joonyoung Jang, Yulhyun Park, Jun Chang Lee.

**Methodology:** Seon Cho.

**Project administration:** Ju Seok Ryu.

**Supervision:** Ju Seok Ryu.

**Writing – original draft:** Joonyoung Jang.

**Writing – review & editing:** Sunmok Hong, Jiwoon Lim, Ju Seok Ryu.
